# Variation in and Factors Associated With US County-Level Cancer Mortality, 2008-2019

**DOI:** 10.1001/jamanetworkopen.2022.30925

**Published:** 2022-09-09

**Authors:** Weichuan Dong, Wyatt P. Bensken, Uriel Kim, Johnie Rose, Qinjin Fan, Nicholas K. Schiltz, Nathan A. Berger, Siran M. Koroukian

**Affiliations:** 1 Department of Population and Quantitative Health Sciences, Case Western Reserve University School of Medicine, Cleveland, Ohio; 2 Kellogg School of Management, Northwestern University, Evanston, Illinois; 3 Case Comprehensive Cancer Center, Case Western Reserve University, Cleveland, Ohio; 4 Center for Community Health Integration, School of Medicine, Case Western Reserve University, Cleveland, Ohio; 5 Surveillance and Health Equity Science, American Cancer Society, Kennesaw, Georgia; 6 Frances Payne Bolton School of Nursing, Case Western Reserve University, Cleveland, Ohio; 7 Center for Science, Health, and Society, School of Medicine, Case Western Reserve University School of Medicine, Cleveland, Ohio

## Abstract

**Importance:**

The association between cancer mortality and risk factors may vary by geography. However, conventional methodological approaches rarely account for this variation.

**Objective:**

To identify geographic variations in the association between risk factors and cancer mortality.

**Design, Setting, and Participants:**

This geospatial cross-sectional study used county-level data from the National Center for Health Statistics for individuals who died of cancer from 2008 to 2019. Risk factor data were obtained from County Health Rankings & Roadmaps, Health Resources and Services Administration, and Centers for Disease Control and Prevention. Analyses were conducted from October 2021 to July 2022.

**Main Outcomes and Measures:**

Conventional random forest models were applied nationwide and by US region, and the geographical random forest model (accounting for local variation of association) was applied to assess associations between a wide range of risk factors and cancer mortality.

**Results:**

The study included 7 179 201 individuals (median age, 70-74 years; 3 409 508 women [47.5%]) who died from cancer in 3108 contiguous US counties during 2008 to 2019. The mean (SD) county-level cancer mortality rate was 177.0 (26.4) deaths per 100 000 people. On the basis of the variable importance measure, the random forest models identified multiple risk factors associated with cancer mortality, including smoking, receipt of Supplemental Nutrition Assistance Program (SNAP) benefits, and obesity. The geographical random forest model further identified risk factors that varied at the county level. For example, receipt of SNAP benefits was a high-importance factor in the Appalachian region, North and South Dakota, and Northern California; smoking was of high importance in Kentucky and Tennessee; and female-headed households were high-importance factors in North and South Dakota. Geographic areas with certain high-importance risk factors did not consistently have a corresponding high prevalence of the same risk factors.

**Conclusions and Relevance:**

In this cross-sectional study, the associations between cancer mortality and risk factors varied by geography in a way that did not correspond strictly to risk factor prevalence. The degree to which other place-specific characteristics, observed and unobserved, modify risk factor effects should be further explored, and this work suggests that risk factor importance may be a preferable paradigm for selecting cancer control interventions compared with risk factor prevalence.

## Key Points

**Question:**

How does the association between cancer mortality and risk factors vary across US counties?

**Findings:**

In this cross-sectional study, the associations between cancer mortality and risk factors, such as smoking or obesity, varied greatly across US counties. Large-scale spatial patterns of variable importance did not necessarily mirror risk factor prevalence patterns.

**Meaning:**

These findings suggest that place-specific associations of risk factors with cancer mortality may represent an alternative to risk factor prevalence for prioritizing cancer control interventions.

## Teaser

This cross-sectional study examines geographic variations in the association between risk factors and cancer mortality.

## Introduction

Although substantial progress has been made in reducing overall cancer mortality, substantial geographic disparities in cancer mortality persist.^1,2^ Traditional cancer prevention and control efforts have been targeted toward communities with a high prevalence of known cancer risk factors, presuming that a high prevalence of these factors directly translates to a higher risk of cancer.^3,4^ In reality, these factors likely have a contextual, place-dependent relationship with health outcomes.^5^ Thus, understanding the geographic association between cancer mortality and risk factors is key to targeting and optimizing resources directed at cancer disparities elimination.

Because of limitations in modeling techniques, investigators have traditionally needed to assume that risk factors affect individuals uniformly across geographies. Although efforts have been made to model place-dependent associations between risk factors and mortality using geographically weighted regression (GWR),^6,7,8^ these models may be limited in evaluating nonlinear relationships and are not well suited to simultaneously evaluate a vast number of variables that may be correlated.^9^

Using a tree-based nonparametric method, the conventional random forest (RF) algorithm can overcome these issues. Such models have been applied in several studies on cancer mortality.^10,11^ A recently developed geographical random forest (GRF) algorithm can now account for the spatially varying association between the outcome and risk factors.^12^ This cross-sectional study aims to describe place-specific risk factors of cancer mortality using the conventional RF (at the national and regional scales) and the GRF (at the local scale at the county level) with the goal of facilitating and targeting intervention efforts.

## Methods

Given the deidentified nature of the data, the Case Western Reserve University institutional review board determined that this cross-sectional study did not constitute human participants research and was thus exempted from review and the need for informed consent, in accordance with 45 CFR §46. This study was conducted from October 2021 to July 2022 and followed the Strengthening the Reporting of Observational Studies in Epidemiology (STROBE) reporting guidelines.

### Data Sources

County-level, age-adjusted cancer mortality rates during 2008 to 2019 came from the National Center for Health Statistics and were accessed through the SEER*Stat software, a resource from the National Cancer Institute.^13^ We obtained county-level risk factor data from multiple sources, including County Health Rankings & Roadmaps,^14^ Area Health Resources Files,^15^ National Cancer Institute,^16^ and Centers for Disease Control and Prevention.^17^ The Table shows the description, year, and source of these variables. Race and ethnicity information was obtained from the County Health Rankings & Roadmaps (original source, Census–Population Estimates) and was analyzed in this study to determine whether other risk factors were more associated with cancer mortality than race and ethnicity. Since the study focuses on cancer mortality at the county level, all measures were aggregated at the county level; therefore, we did not account for individual-level risk factors.

**Table.** County-Level Demographic and Risk Factor Variables

Source (year published) and variable	Description	Year of data	Original source
County Health Rankings & Roadmaps (2016)			
Non-Hispanic Black	Percentage of Non-Hispanic African American people	2014	Census-PE
Hispanic population	Percentage of Hispanic people	2014	Census-PE
Population aged ≥65 y	Percentage of people aged ≥65 y	2014	Census-PE
Median household income	The income where half of households in a county earn more and half of households earn less	2014	Census-SAIPE
Unemployment	Percentage of population (aged ≥16 y) unemployed but seeking work	2014	BLS
Smoking	Percentage of adults who are current smokers (age-adjusted)	2014	CDC-BRFSS
Excessive drinking	Percentage of adults reporting binge or heavy drinking (age-adjusted)	2014	CDC-BRFSS
Poor or fair health	Percentage of adults reporting fair or poor health (age-adjusted)	2014	CDC-BRFSS
Frequent physical distress	Percentage of adults reporting ≥14 d of poor physical health per month (age-adjusted)	2014	CDC-BRFSS
Frequent mental distress	Percentage of adults reporting ≥14 d of poor mental health per month (age-adjusted)	2014	CDC-BRFSS
Not proficient in English	Percentage of people (aged ≥5 y) who reported speaking English less than very well	2010-2014	Census-ACS
County Health Rankings & Roadmaps (2017)			
Rural population	Percentage of people living in rural areas	2010	Census-PE
Physical inactivity	Percentage of adults (aged ≥18 y) reporting no leisure-time physical activity (age-adjusted)	2013	CDC-DIA
Adult obesity	Percentage of the adult population (aged ≥18 y) that reports a body mass index ≥30 (age-adjusted)^a^	2013	CDC-DIA
Diabetes	Percentage of adults (aged ≥20 y) with diagnosed diabetes (age-adjusted)	2013	CDC-DIA
Uninsured adults	Percentage of adults aged <65 y without health insurance	2014	Census-SAHIE
Social associations	Number of membership associations per 10 000 population	2014	Census-CBP
Insufficient sleep	Percentage of adults who report <7 h of sleep on average (age-adjusted)	2014	CDC-BRFSS
Sexually transmitted infections	Number of newly diagnosed chlamydia cases per 100 000 population	2014	CDC-NCHHSTP
Preventable hospital stays	Rate of hospital stays for ambulatory-care sensitive conditions per 100 000 Medicare enrollees	2014	DAHC
Health care costs	Per capita spending of Medicare enrollees	2014	DAHC
Food environment index	Index of factors that contribute to a healthy food environment, from 0 (worst) to 10 (best)	2014	USDA-FEA
Mammography use (aged 65-69 y)	Percentage of female Medicare enrollees (aged 65-69 y) who received an annual mammography screening	2014	DAHC
Severe housing problems	Percentage of households with at least 1 of 4 housing problems: overcrowding, high housing costs, lack of kitchen facilities, or lack of plumbing facilities	2009-2013	HUD-CHAS
Access to exercise opportunities	Percentage of population with adequate access to locations for physical activity	2010 & 2014	ESRI and Census (TF)
Violent crime	Number of reported violent crime offenses per 100 000 population	2012-2014	FBI-UCR
Income inequality	Ratio of household income at the 80th percentile to income at the 20th percentile	2011-2015	Census-ACS
Children in single-parent households	Percentage of children who live in a household headed by a single parent	2011-2015	Census-ACS
Driving alone to work	Percentage of the workforce that drives alone to work	2011-2015	Census-ACS
Long commute, driving alone	Among workers who commute in their car alone, the percentage who commute >30 min	2011-2015	Census-ACS
HRSA–Area Health Resources Files (2019)			
Female-headed households	Percentage of female-headed households	2010	Census (decennial)
Rural-urban continuum code	An ordinal variable classifying metropolitan and nonmetropolitan counties by the population size of their metropolitan area and by degree of urbanization and adjacency to a metropolitan area or areas	2013	USDA-ERS
Urban influence code	An ordinal variable classifying metropolitan and nonmetropolitan counties by the population size of their metropolitan area and by size of the largest city or town and proximity to metropolitan and micropolitan areas	2013	USDA-ERS
Poverty	Percentage of people in poverty	2014	Census-SAIPE
Income <200% of federal poverty level	Percentage of people (aged 18-64 y) with income <200% of federal poverty level	2014	Census-SAHIE
Receipt of SNAP benefits	Percentage of households with ≥1 individual who received SNAP benefits	2014	Census-SNAPF
Medicare eligibility	Percentage of people eligible for Medicare	2014	CMS
Primary care physicians	Primary care physicians in patient care per 100 000 people	2014	AMA
Obstetrician-gynecologists	Obstetrician-gynecologists in patient care per 100 000 people	2015	AMA
Radiation oncologists	Radiation oncologists per 100 000 people	2015	AMA
Radiologists	Diagnostic radiologists in patient care per 100 000 people	2015	AMA
Hospitals	Hospitals per 100 000 people	2015	AMA
Community health centers	Community health centers per 100 000 people	2014	HRSA
Health professional shortage area	An ordinal variable identifying counties experiencing a shortage of health professionals (primary care physicians)	2015	HRSA
Without high school degree	Percentage of people (aged ≥25 y) with no high school diploma	2011-2015	Census-ACS
National Cancer Institute (2016)			
Mammography use (aged ≥40 y)	Percentage of female individuals (aged ≥40 y) who received a mammography screening within 2 y	2008-2010	CDC-BRFSS and NHIS
Colorectal screening (aged ≥50 y)	Percentage of people (aged ≥50 y) who ever had colorectal cancer test (home-based fecal occult blood test in the past 2 y or ever had a colorectal endoscopy)	2008-2010	CDC-BRFSS and NHIS
CDC Division for Heart Disease and Stroke Prevention (2020)			
Cardiovascular disease	Total cardiovascular disease hospitalization rate per 1000 Medicare beneficiaries (aged ≥65 y)	2013-2015	CMS
Stroke	Stroke hospitalization rate per 1000 Medicare beneficiaries (aged ≥65 y)	2013-2015	CMS

Abbreviations: ACS, American Community Survey; AMA, American Medical Association; BLS, Bureau of Labor Statistics; BRFSS, Behavioral Risk Factor Surveillance System; CBP, County Business Patterns; CDC, Centers for Disease Control and Prevention; CHAS, Comprehensive Housing Affordability Strategy data; CMS, Centers for Medicare & Medicaid Services; DAHC, Dartmouth Atlas of Health Care; DIA, Diabetes Interactive Atlas; ERS, Economic Research Service; FEA, Food Environment Atlas; HRSA, Health Resources and Services Administration; HUD, US Department of Housing and Urban Development; NCHHSTP, National Center for HIV/AIDS, Viral Hepatitis, STD, and TB prevention; NHIS, National Health Interview Survey; PE, Population Estimates; SAHIE, Small Area Health Insurance Estimates; SNAP, Supplemental Nutrition Assistance Program; SNAPF, Supplemental Nutrition Assistance Program File; TF, Tigerline File; UCR, Uniform Crime Reporting; USDA, US Department of Agriculture.

^a^ Body mass index is calculated as weight in kilograms divided by height in meters squared.

### Study Population

The study included individuals who died from any cancer in the contiguous US during 2008 to 2019. The cancer mortality rates for counties with fewer than 11 cancer deaths during the study period were imputed using the average rates of adjacent counties (ie, counties sharing a common edge or a common vertex with the current county), as is conventional in geographic research.^18^

### Statistical Analysis

The goal of this study was to evaluate the variable importance (VI) measure of risk factors associated with cancer mortality across the contiguous US counties using RF and GRF. VI was based on node impurity, which reflects the extent to which a given variable contributes to decreasing the variance of responses in the RF regression trees. We hypothesized that risk factors had a place-specific association with cancer mortality, and, thus, different sets of important risk factors would be identified at the national, regional, and local scales. To achieve this goal, we conducted the conventional RF nationwide (national-RF) and in 4 US regions (regional-RF). We also conducted GRF accounting for the local variation across US counties. We note that VI was used to identify the most relevant markers of cancer risk rather than to establish their role in any causal relationship.

The conventional RF is a tree-based, nonlinear, nonparametric machine learning method that creates multiple classification and regression trees^19^ using random variable selection and bootstrap aggregation methods.^20^ The results include a VI plot with the most important variable at the top and set to 100%, and the rest of the variables scaled according to their importance relative to the most important variable. The study areas of the RF models were the contiguous US for the national-RF and 4 separate regions of the country (Northeast, Midwest, West, and South)^21^ for the regional-RF. To improve the performance of the RF models, we adopted a variable selection algorithm that selected risk factors having the smallest out-of-bag error in the model prediction, as suggested in previous studies.^22,23^

The GRF is an RF-based model with the assumption that the true underlying associations between the outcome and risk factors vary geographically.^12^ The GRF is loosely based on the concept of spatially varying coefficient models, such as the GWR.^5,24,25^ In contrast to the conventional RF in which results are drawn from the whole extent of the study area, the GRF conducts multiple RFs on varying subregions of the study area. Each subregion includes a target county and a predefined number of nearest counties. We illustrated a hypothetical example of a subregion of the GRF consisting of the target county and its 50 nearest neighbors in the Northeast US in eFigure 1 in the Supplement. Since every county in the study area can serve as a target county, the number of subregions is equal to the number of counties in the study area. Thus, the GRF in this study included 3108 submodels. As a result, there will be a measure of VI for each variable in each submodel. Finally, the distribution of VI of any risk factor can be visualized on a map, with counties representing their respective submodels.

Increasing the number of counties included in submodels results in a tradeoff between model performance (as determined by the local pseudo coefficient of determination, or *R*^2^)^12^ and the extent of local representation. Therefore, we tested the GRF with 50, 100, 200, and 400 nearest counties and chose the parameter that optimized the balance between the overall model performance and the extent of local representation of the submodels.

Both the conventional RF and the GRF included all risk factors listed in the Table. All models included 2000 trees and 6 variables randomly sampled as candidates at each split to optimize model performance and computation time in our study.

SAS statistical software version 9.4 (SAS Institute) and R statistical software version 4.2.1 (R Project for Statistical Computing) were used for the analyses, and ArcGIS Pro software version 2.9.0 (Esri) was used for mapping. The R packages VSURF version 1.1.0^23^ and randomForest version 4.7-1.1^26^ were used for variable selection and conventional RF, and SpatialML version 0.1.4^27^ was used for GRF.

## Results

**Figure 1.**
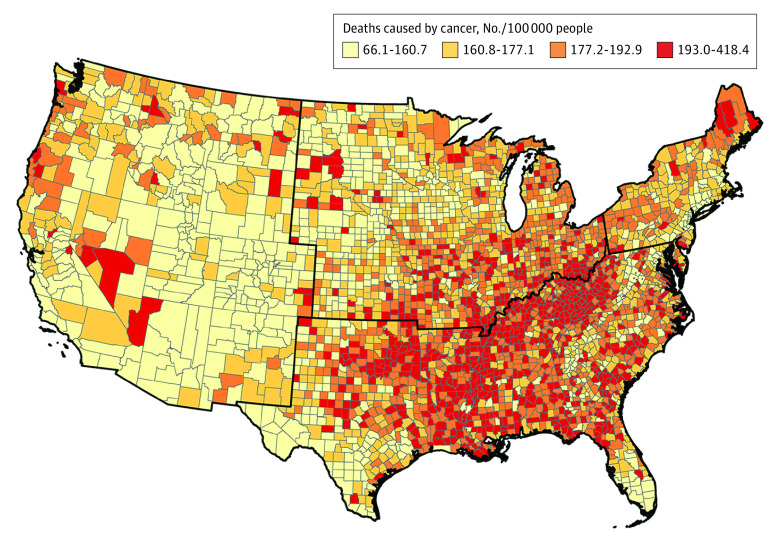
US County-Level Cancer Mortality Rate by Quartile During 2008 to 2019 Black lines delineate regions of the US (Northeast, Midwest, South, and West).

During 2008 to 2019, 7 179 201 people in the 3018 contiguous US counties died of cancer, of whom 6 128 781 (85.4%) were White, 834 491 (11.6%) were Black, 179 660 (2.5%) were Asian or Pacific Islander, and 36 269 (0.6%) were American Indian/Alaska Native. The median age of persons who died from cancer was 70 to 74 years (SEER*Stat reports age in 5-year increments), and women accounted for 47.5% of the deaths (3 409 508 women). Figure 1 illustrates the county-level cancer mortality per 100 000 people in the contiguous US, ranging from 66.1 deaths per 100 000 people in Summit County, Colorado (178 deaths), to 418.4 deaths per 100 000 people in Union County, Florida (873 deaths). The median (IQR) and mean (SD) of county-level cancer mortality rates were 177.0 (160.7-192.8) and 177.0 (26.4) deaths per 100 000 people, respectively, and 95% of counties had cancer mortality rates ranging from 125.3 to 230.0 deaths per 100 000 people. The highest cancer mortality rates were mostly in the South. The mortality rates for 4 counties (Banner and Thomas counties in Nebraska, and King and Loving Counties in Texas) with fewer than 11 cancer deaths were imputed by the average rate of their adjacent counties.

**Figure 2.**
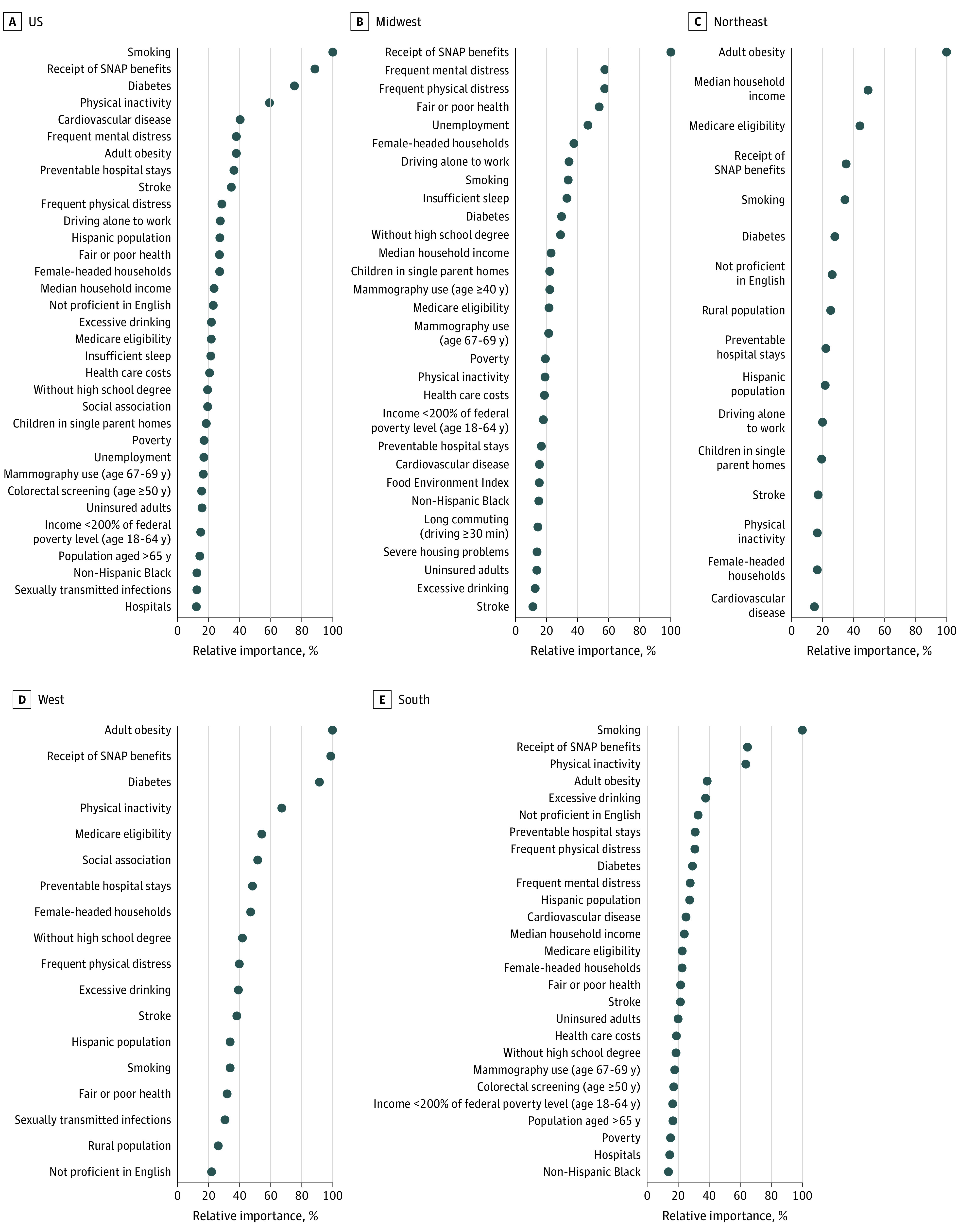
Relative Importance Plot of Cancer Mortality Risk Factors From the Conventional Random Forest Models The most important variable is at the top and set to 100%. The importance of the rest of the variables is shown relative to the top one. In panel B, states included in Midwest region were IN, IA, IL, KS, MI, MO, MN, NE, ND, OH, SD, and WI. In panel C, states included in the Northeast region were CT, ME, MA, NH, NJ, NY, PA, RI, and VT. In panel D, states included in West region were AZ, CA, CO, ID, MT, NV, NM, OR, UT, WA, and WY. In panel E, states included in South region were AL, AR, DE, DC, FL, GA, KY, LA, MD, MS, NC, OK, SC, TN, TX, VA, and WV. SNAP indicates Supplemental Nutrition Assistance Program.

The variable selection algorithm selected 33 of the 49 variables for the national-RF model, with smoking, receipt of Supplemental Nutrition Assistance Program (SNAP) benefits, diabetes, and physical inactivity as the top 4 in order of VI (Figure 2). In contrast, the regional-RF selected 16 variables for the Northeast, 29 variables for the Midwest, 27 variables for the South, and 18 variables for the West (Figure 2). Smoking and receipt of SNAP benefits were ranked first in the South and the Midwest, respectively. The results of the regional-RF also suggested substantial differences in the VI among risk factors in different regions. For example, despite adult obesity being the top-ranking variable in the Northeast and the West regional-RF models, it was only ranked seventh in the national-RF and was not selected by the Midwest regional-RF. On the other hand, variables such as diabetes and physical inactivity, which were deemed of high VI in the national-RF, had low VI in some of the regional-RF models.

The comparisons of GRF models with different numbers of neighboring counties is shown in the eTable in the Supplement. The mean (SD) pseudo *R*^2^ of the submodels increased from 26.8% (21.1%) to 34.2% (18.1%) when the number of nearest neighbors increased from 50 to 100. The mean (SD) pseudo *R*^2^ further increased to 40.6% (15.0%) and 45.7% (12.2%) when the number of neighbors was 200 and 400, respectively. Comparing the 200-neighbor and 400-neighbor models, the performance increased by approximately 5 percentage points in the mean pseudo *R*^2^, whereas the extent of local representation decreased by half. Therefore, we decided to adopt the 200-neighbor GRF as our main model.

**Figure 3.**
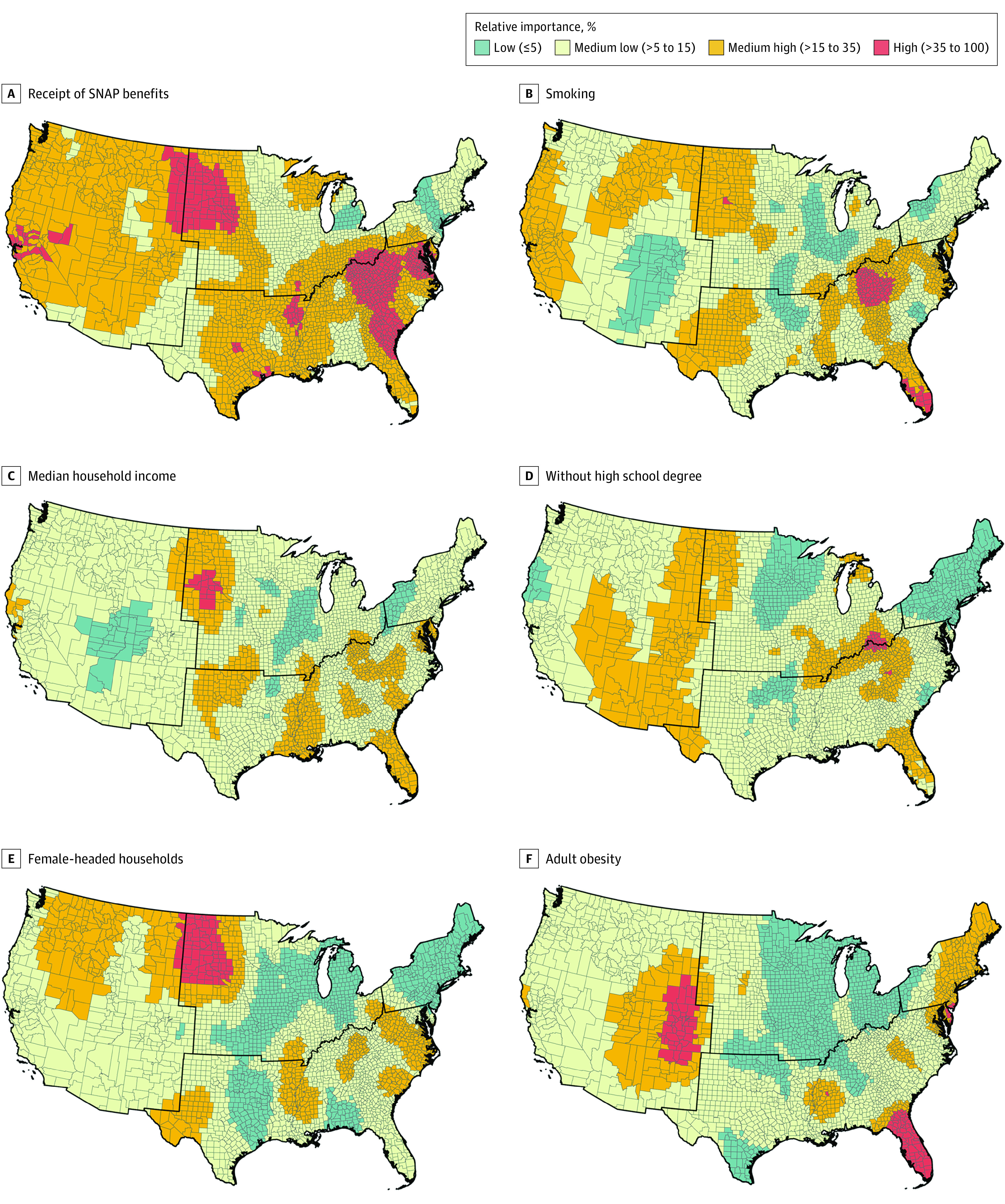
Relative Importance of Selected Cancer Risk Factors From the Geographical Random Forest Analysis The highest value of variable importance among all risk factors is set to 100%. All other values of variable importance are scaled relative to the highest value. Black lines delineate regions of the US (Northeast, Midwest, South, and West). SNAP indicates Supplemental Nutrition Assistance Program.

From the results of our main GRF model, we visualized the spatial distribution of VI for the top-6 risk factors according to their average VI of submodels (Figure 3), including receipt of SNAP benefits, smoking, median household income, high school degree, female-headed households, and adult obesity. Individual high-resolution maps in Figure 3 are provided in eFigure 2 in the Supplement. The highest value of VI among all risk factors is set to 100% and all other values of VI are scaled relative to the highest value. The performance of the GRF is indicated by the local pseudo *R*^2^, with values ranging from 0% to 74% across all counties (eFigure 3 in the Supplement), where submodels in the non-Appalachian Southeast and some plains states had the lowest performance.

In Figure 3, we note that high or medium-high VI for receipt of SNAP benefits was observed in more than two-thirds of the counties in the GRF, especially around the northeastern part of the South, North and South Dakota, Nebraska, Northern California, and a few counties in Arkansas and Texas. Counties with high smoking VI were observed in Kentucky and Tennessee, as well as in southern Florida, whereas many counties in the Great Lakes region and the Mountain States had low smoking VI. Median household income was of high VI in many South Dakota and Nebraska counties. High or medium-high VI of high school degree was observed in counties along the Ohio River, Appalachian counties, some Western states, and Florida. Female-headed household was of high VI in North and South Dakota. Adult obesity also had a large geographic variation of VI, where most counties with high or medium-high VI were clustered around Colorado, Florida, the Northeast, and Mississippi.

**Figure 4.**
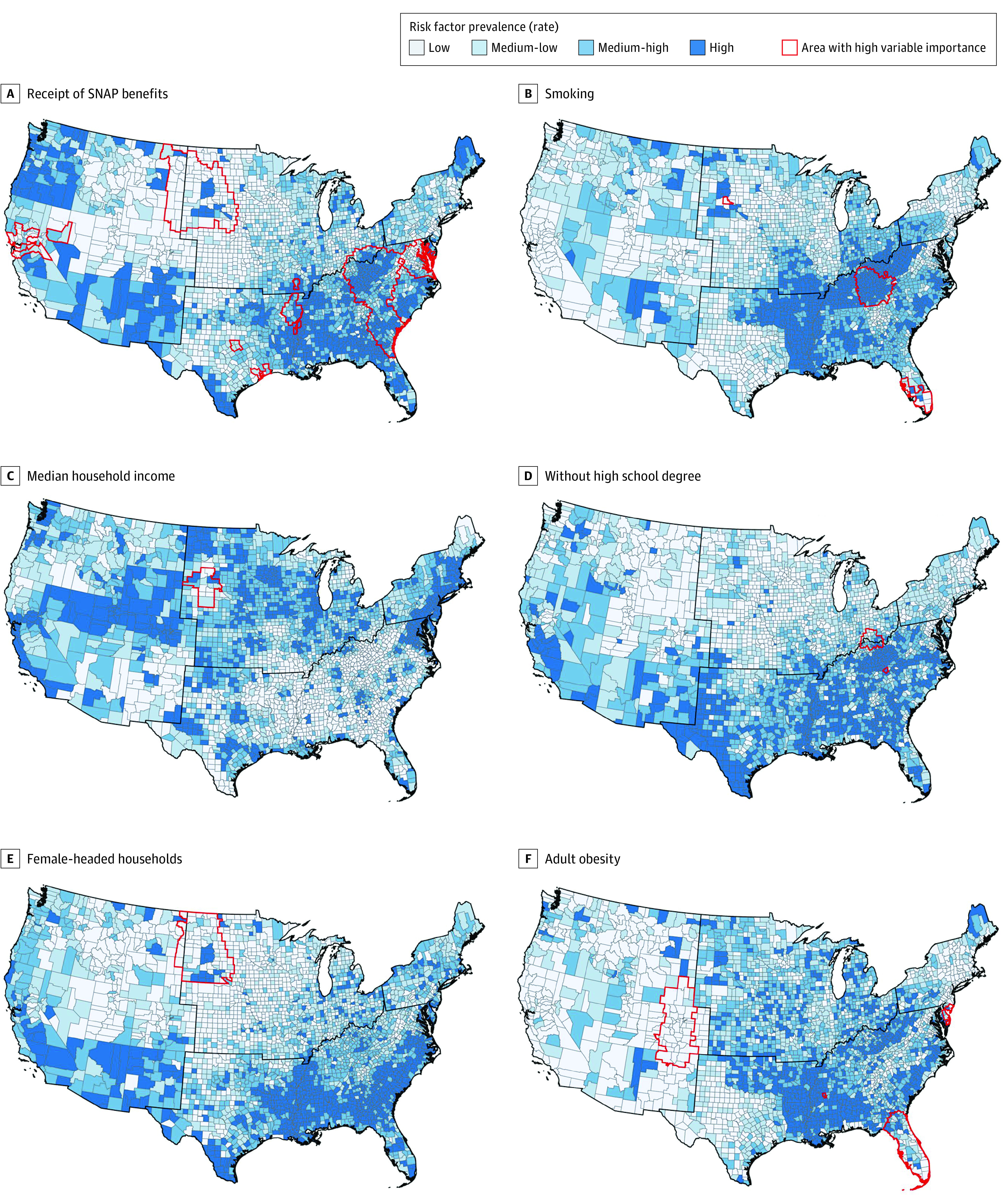
Risk Factors Prevalence and Areas With High Variable Importance From the Geographical Random Forest Analysis All variables use their own respective scales and are classified by quartile. Black lines delineate regions of the US (Northeast, Midwest, South, and West). SNAP indicates Supplemental Nutrition Assistance Program.

Figure 4 shows the prevalence maps of the 6 risk factors presented in Figure 3. Except for median household income, for which the majority of high-prevalence counties were observed in the Northeast coastal area, the Midwest, and the West, receipt of SNAP benefits, smoking, high school degree, female-headed households, and adult obesity were highly prevalent in the South. Individual high-resolution maps in Figure 4 are provided in eFigure 4 in the Supplement.

We overlaid geographic areas with high VI risk factors on risk factor prevalence maps in Figure 4. Some areas with a high VI of certain risk factors also corresponded to a high prevalence, such as receipt of SNAP benefits in the Appalachian region and smoking in Kentucky and Tennessee. In other places, however, the opposite (ie, high VI and low prevalence) was observed, such as the western border of the Midwest for receipt of SNAP benefits and female-headed households, and counties around Colorado for adult obesity.

## Discussion

Using conventional RF, this cross-sectional study analyzed the importance of county-level risk factors associated with cancer mortality in the US. The GRF further uncovered the spatially varying associations between risk factors and cancer mortality at the county level, association that the conventional RF could not detect.

Among all risk factors, receipt of SNAP benefits had the strongest VI in association with cancer mortality in many parts of the US, which is consistent with a previous study^28^ linking receipt of SNAP benefits with increased tumor size at the time of diagnosis. In fact, in addition to being an indicator of low income, a high number of individuals receiving SNAP benefits in a given area has been shown to be a strong marker for limited access to healthy foods and higher risk of obesity.^29,30,31^ On the other hand, poverty was not identified as an important factor associated with cancer mortality in the current study according to the variable rankings in Figure 2. Thus, it is still unclear which characteristics of being a recipient of SNAP benefits have contributed to a higher risk of cancer mortality. Future studies should elucidate the differences between receipt of SNAP benefits and poverty relative to cancer incidence and mortality so that specific factors associated with adverse cancer outcomes can be targeted more effectively.

Other important variables included smoking, diabetes, median household income, high school degree, female-headed households, and adult obesity. Among these, smoking and obesity were established modifiable behavioral or metabolic risk factors,^32,33,34^ where interventions, such as smoking cessation and weight control programs, could be targeted in geographic areas with high VI. Additionally, our study further affirms that socioeconomic status variables, such as income, education, and female-headed households, are associated with cancer mortality as suggested in previous studies.^35,36,37,38^

Figure 4 demonstrates that high risk factor prevalence does not consistently correspond to high VI. For example, although obesity prevalence was generally higher in the South compared with that in the West, obesity VI in several western states around Colorado was higher than in most parts of the South except for Florida. Indeed, the lack of correspondence in some locations between prevalence and VI suggests that cancer mortality risk factors may be modified by place-specific factors.^38,39,40,41^ It is conceivable that the interplay of a given risk factor with comorbidities or other exposures, observed or unobserved, may potentiate or ameliorate the association of that risk factor. Thus, the difference between prevalence and VI might be associated with modulating factors, either positive or negative, where further investigations are needed. Nevertheless, this work suggests that compared with prevalence, risk factor importance in a given geographic area may be preferred in selecting cancer control interventions.

Our study has several advantages. First, compared with existing works that used only the nonspatial conventional RF or the linear GWR on cancer mortality,^6,7,8,10,11^ our study used the GRF, which accounted for both spatially varying and nonlinear associations between cancer mortality and risk factors. Previous applications of GRF to other health outcomes have also demonstrated its superior performance over RF and GWR models.^42,43^ This study further demonstrates the utility of GRF in cancer epidemiology. Furthermore, our study uncovered discordance between the rankings of prevalence and the rankings of importance of cancer risk factors in certain geographic areas, which suggests the need for further investigations on effect modification of cancer risks.

### Limitations

One limitation of this study is that we did not account for cancer incidence and tumor-specific characteristics such as cancer type and stage at diagnosis in cancer mortality. However, using cancer-specific mortality as the outcome has the advantage of capturing a broad span of risk factors that could contribute to cancer death across the cancer prevention and control continuum, including health behaviors and socioeconomic characteristics, demographic and environmental factors, comorbidity prevalence, cancer screenings, and receipt of treatment and survivorship care.

A second limitation is that counties are relatively large units where population characteristics may vary greatly within a county. However, smaller units, such as Census tracts, often introduce the small number problem where the data for less populated areas would need to be suppressed to protect patient confidentiality and to ensure statistical stability. A third limitation is that like other spatial analysis techniques that produce localized and mappable outputs, GRF is subject to edge effects, where target counties close to the edge of the study area are not at the center of their nearest neighbors, generating some degree of bias affecting coastal areas.^44^

A fourth limitation is that data for many risk factors were collected from surveys based on a sample of target populations, where response rates and selection bias could impact the validity of the measures.^45^ Additionally, we did not consider the effect of temporal changes of risk factors in which they could have latent effects on cancer death, resulting in inaccurate measures of their associations.

A fifth limitation is that the focus here is not to discover causal pathways for cancer mortality, noting that causality cannot be established from this study, given its cross-sectional nature. Additional information needs to be considered, including whether a given factor has a causal relationship with the outcome and whether that factor is modifiable or nonmodifiable. On the other hand, evaluating the association between area-level risk factors and outcomes is an essential first step toward establishing causality.^46^ The GRF method presented in this study provides a way to consider numerous risk factors simultaneously, while accounting for spatially varying relationships between the outcome and risk factors.

## Conclusions

This study provides a framework for prioritizing efforts in reducing cancer mortality by considering geographic variations in the importance of risk factors. Identifying risk factor profiles that are specific to geographic areas is imperative to monitoring the landscape of cancer mortality and to uncovering disparities along multiple dimensions of population health. Practitioners and policy makers should consider tailored interventions in reducing cancer mortality based on not only the prevalence but also the importance of the place-specific risk factors.

## Notes

Supplement. **eFigure 1.** An Example of the Geographical Random Forest With a Target County and 50 Nearest Neighbors in the Northeast of the United States **eTable.** Performance of Geographical Random Forest Models Comparing Different Numbers of Nearest Neighbors **eFigure 2.** Relative Importance of Selected Cancer Risk Factors From the Geographical Random Forest Analysis (High Resolution Maps) **eFigure 3.** Pseudo Coefficient of Determination (Pseudo *R*^2^) of Counties in Predicting Cancer Mortality From the Geographical Random Forest Model **eFigure 4.** Risk Factors Prevalence and Areas With High Variable Importance From the Geographical Random Forest (High Resolution Maps)
