# Supplementary material for: Variation in and Factors Associated With US County-Level Cancer Mortality, 2008-2019
Source: JAMA Netw Open. 2022 Sep 9;5(9):e2230925. doi: 10.1001/jamanetworkopen.2022.30925 (PMC9463612; doi:10.1001/jamanetworkopen.2022.30925)
Supplement: Supplement. — eFigure 1. An Example of the Geographical Random Forest With a Target County and 50 Nearest Neighbors in the Northeast of the United States eTable. Performance of Geographical Random Forest Models Comparing Different Numbers of Nearest Neighbors eFigure 2. Relative Importance of Selected Cancer Risk Factors From the Geographical Random Forest Analysis (High Resolution Maps) eFigure 3. Pseudo Coefficient of Determination (Pseudo R2) of Counties in Predicting Cancer Mortality From the Geographical Random Forest Model eFigure 4. Risk Factors Prevalence and Areas With High Variable Importance From the Geographical Random Forest (High Resolution Maps) [file jamanetwopen-e2230925-s001.pdf]

Supplemental Online Content

Dong W, Bensken WP, Kim U, et al. Variation in and factors associated with US county-level cancer mortality, 2008-2019. *JAMA Netw Open*. 2022;5(9):e2230925. doi:10.1001/jamanetworkopen.2022.30925

eFigure 1. An Example of the Geographical Random Forest With a Target County and 50 Nearest Neighbors in the Northeast of the United States

eTable. Performance of Geographical Random Forest Models Comparing Different Numbers of Nearest Neighbors

eFigure 2. Relative Importance of Selected Cancer Risk Factors From the Geographical Random Forest Analysis (High Resolution Maps)

eFigure 3. Pseudo Coefficient of Determination (Pseudo *R*2) of Counties in Predicting Cancer Mortality From the Geographical Random Forest Model

eFigure 4. Risk Factors Prevalence and Areas With High Variable Importance From the Geographical Random Forest (High Resolution Maps)

This supplemental material has been provided by the authors to give readers additional information about their work.

© 2022 Dong W et al. *JAMA Network Open.*

eFigure 1. An Example of the Geographical Random Forest With a Target County and 50 Nearest Neighbors in the Northeast of the United States

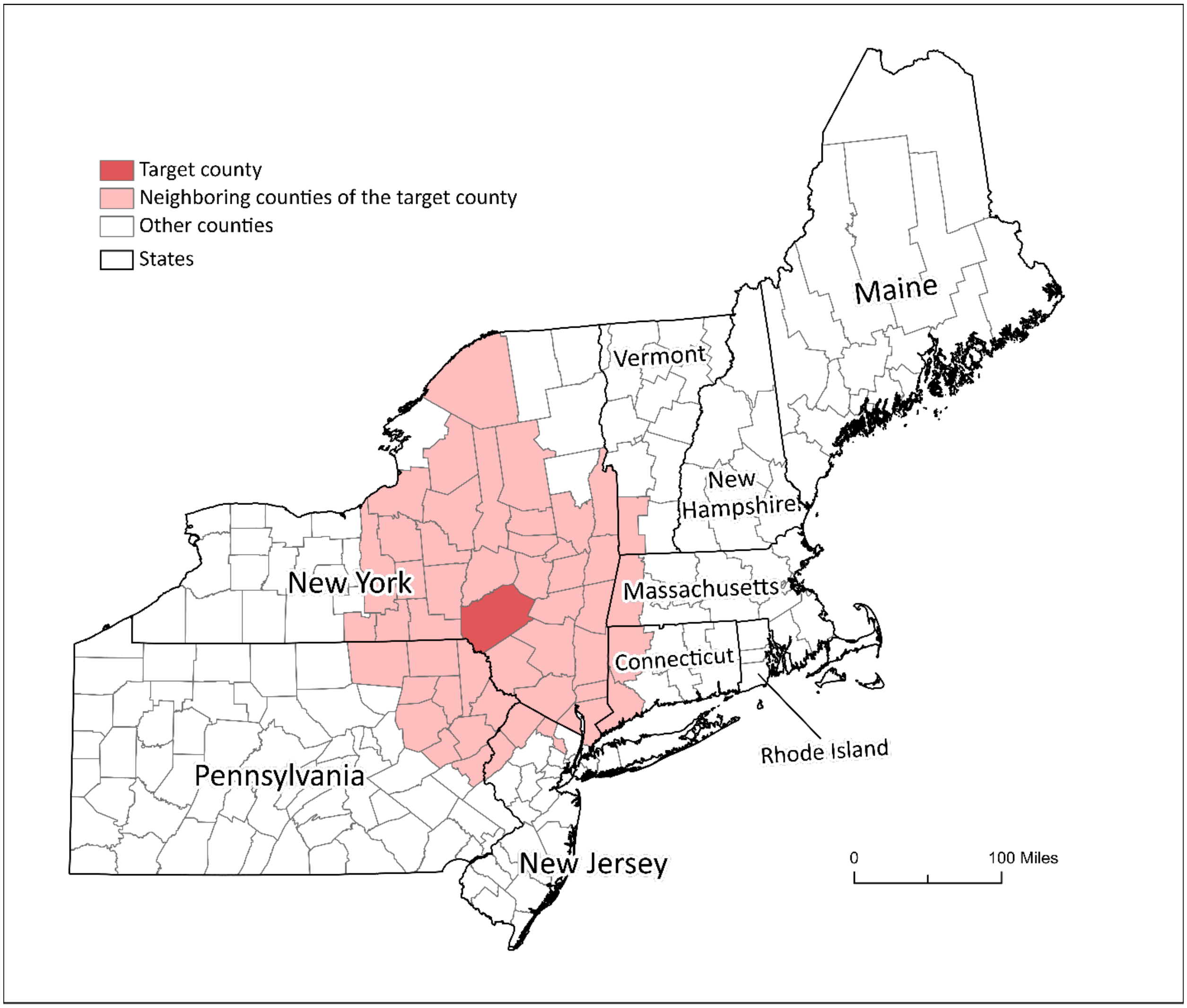

© 2022 Dong W et al. *JAMA Network Open.*

eTable. Performance of Geographical Random Forest Models Comparing Different Numbers of Nearest Neighbors

	Number of nearest neighbors of a target county			
	50	100	200	400
Risk factors*				
1	Receipt of food stamps	Receipt of food stamps	Receipt of food stamps	Receipt of food stamps
2	Median household income	Smoking	Smoking	Smoking
3	Smoking	Median Household Income	Median Household Income	Physical Inactivity
4	Without high school degree	Adult obesity	Without high school degree	Median Household Income
5	Adult obesity	Without high school degree	Female-headed households	Frequent physical distress
6	Preventable hospital stays	Female-headed households	Adult obesity	Fair or poor health
Average Pseudo R^2 (SD) of sub-models	26.8% (21.1%)	34.2% (18.1%)	40.6% (15.0%)	45.7% (12.2%)
Note: The order of risk factors is based on the average value of variable importance measures of sub-models.				

© 2022 Dong W et al. *JAMA Network Open.*

eFigure 2. Relative Importance of Selected Cancer Risk Factors From the Geographical Random Forest Analysis (High Resolution Maps)

Notes: The highest value of variable importance among all risk factors is set to 100%. All other values of variable importance are scaled relative to the highest value.

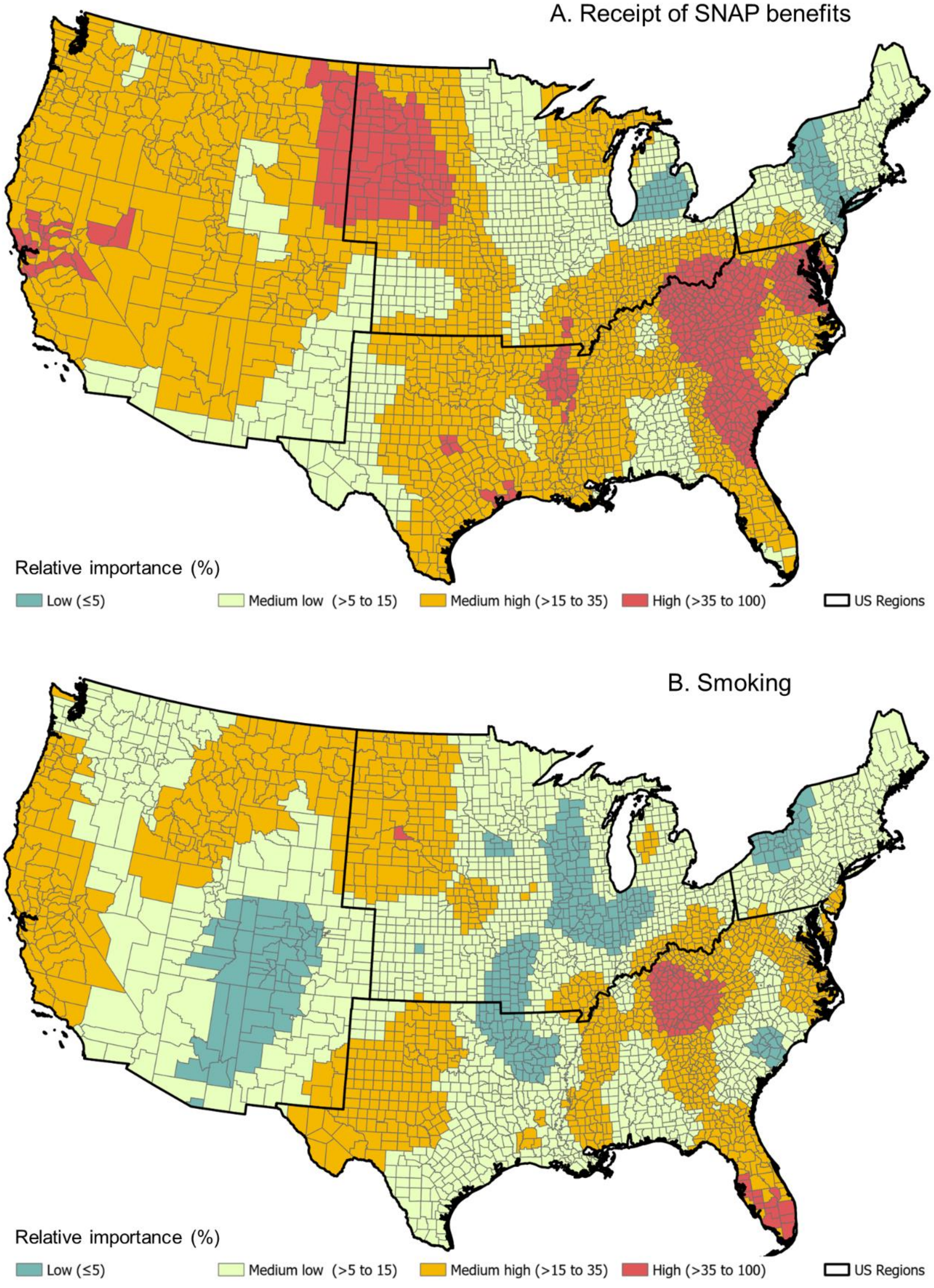

© 2022 Dong W et al. *JAMA Network Open.*

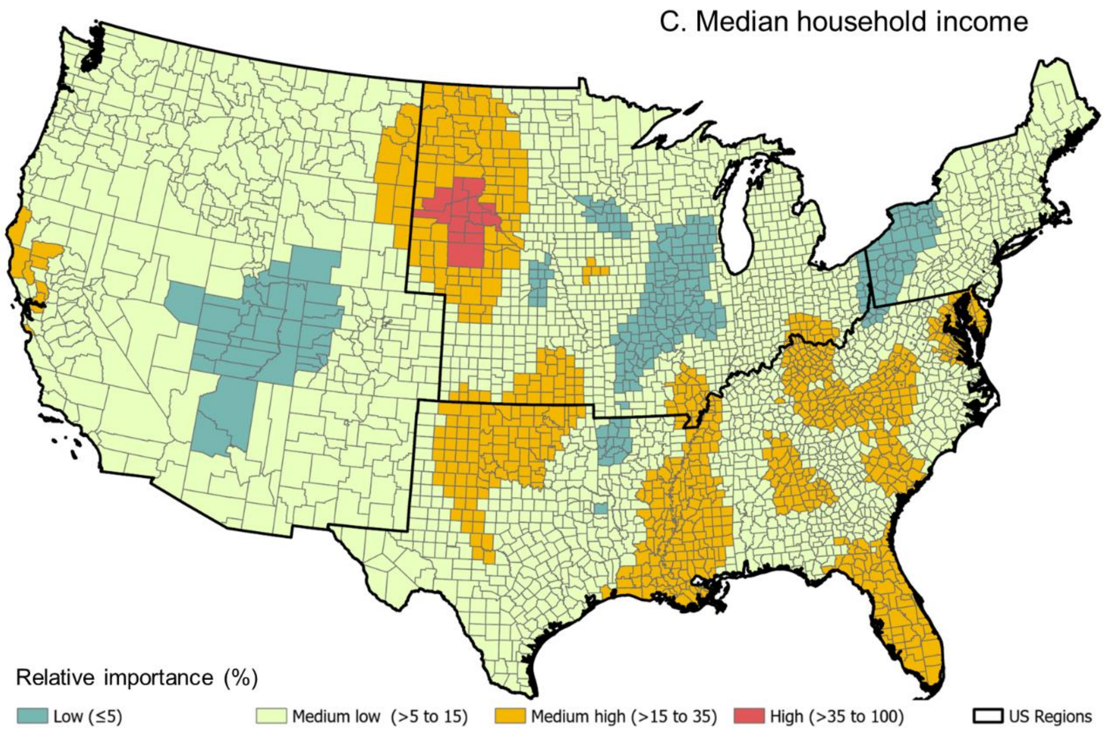

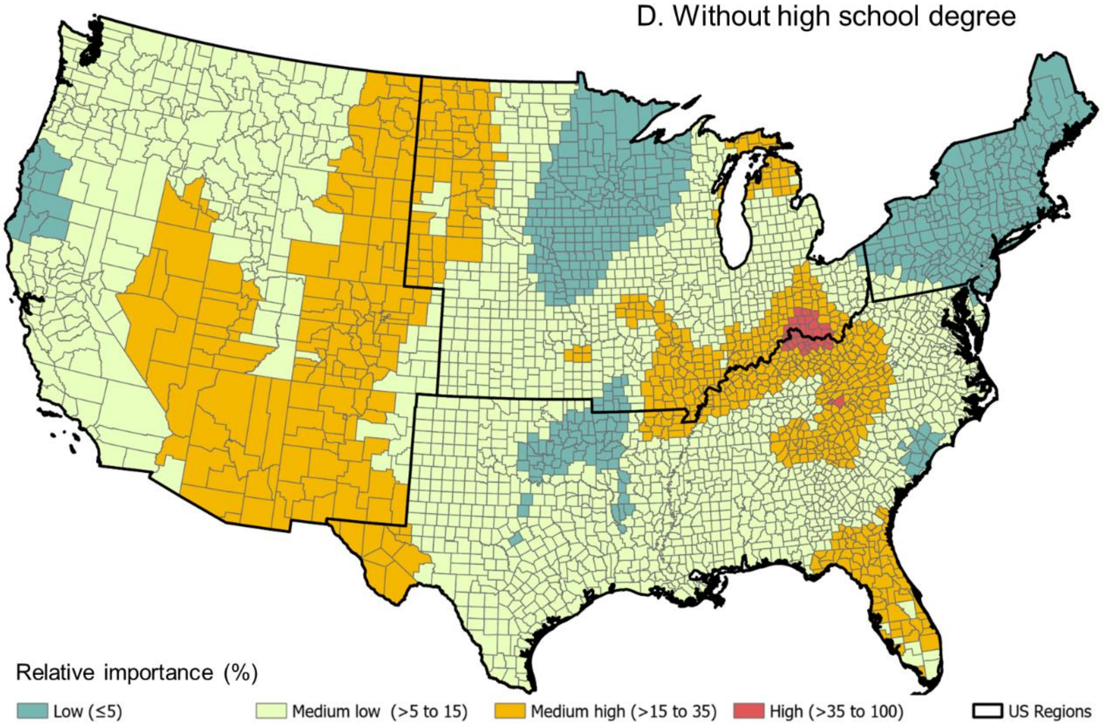

© 2022 Dong W et al. *JAMA Network Open.*

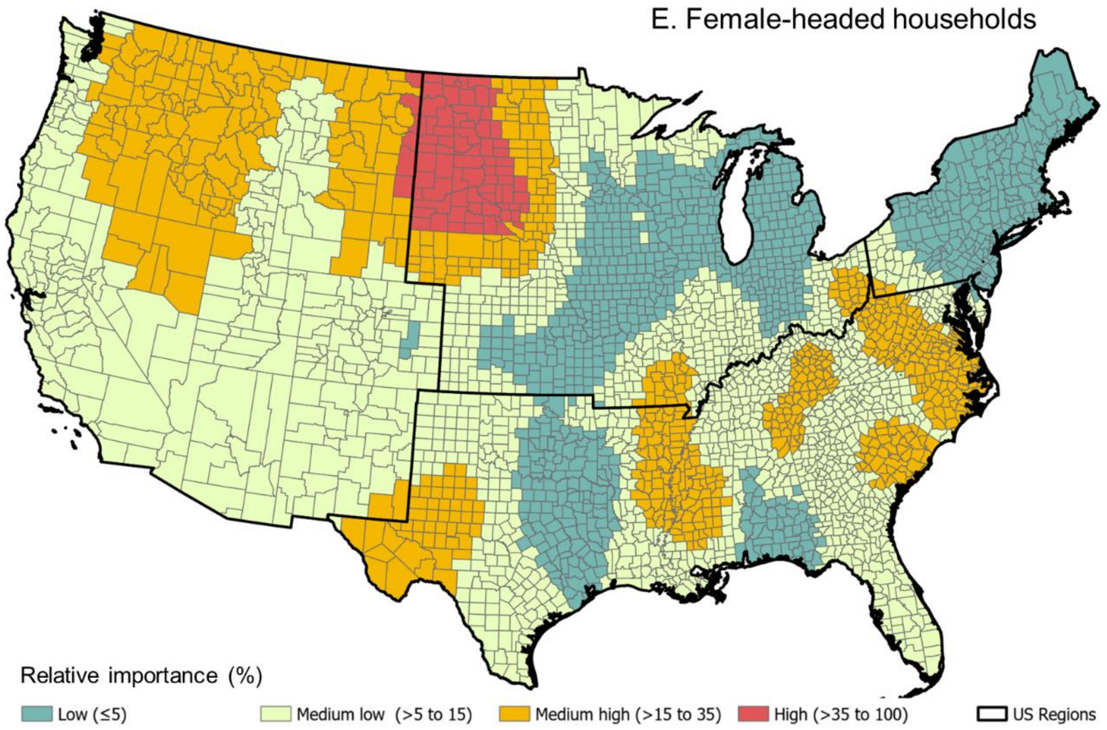

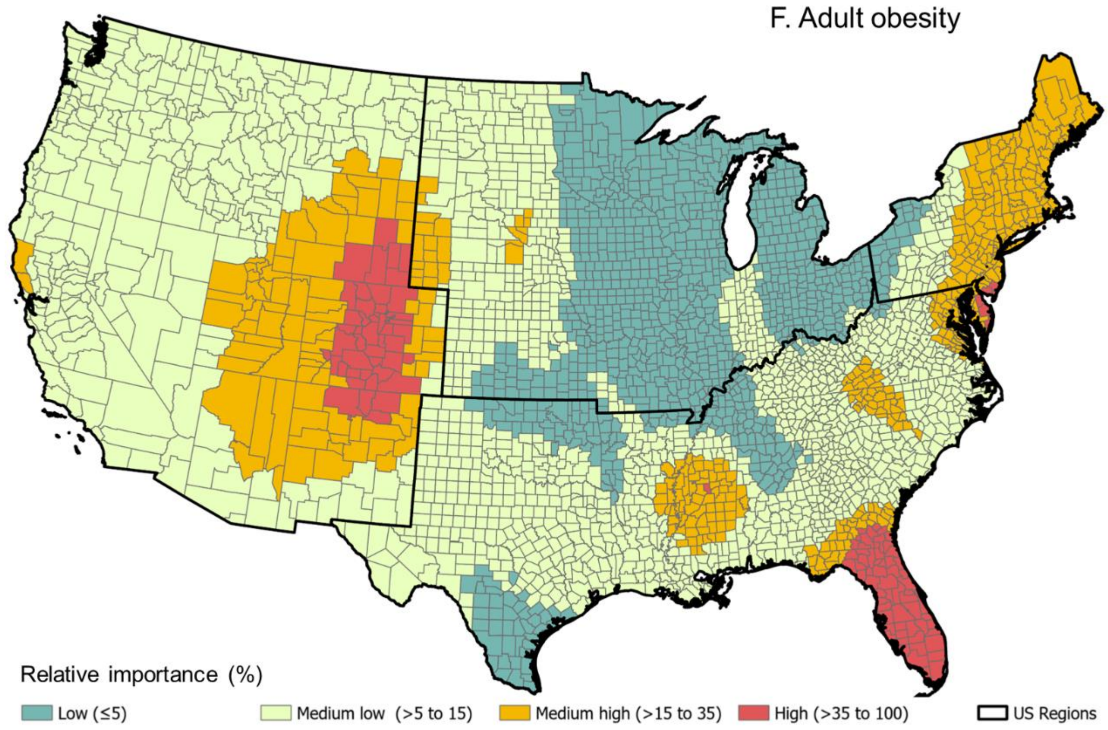

© 2022 Dong W et al. *JAMA Network Open.*

eFigure 3. Pseudo Coefficient of Determination (Pseudo R^2) of Counties in Predicting Cancer Mortality From the Geographical Random Forest Model

Notes: Most areas in the Northeast and the West have relatively high model performance. Areas in the South have both the highest (Kentucky, Tennessee, West Virginia, Virginia, and North Carolina) and the lowest (Mississippi, Alabama, Georgia, and Florida) model performance. In the Midwest, areas in the eastern part mostly have high performance, while those in the western part have relatively low performance.

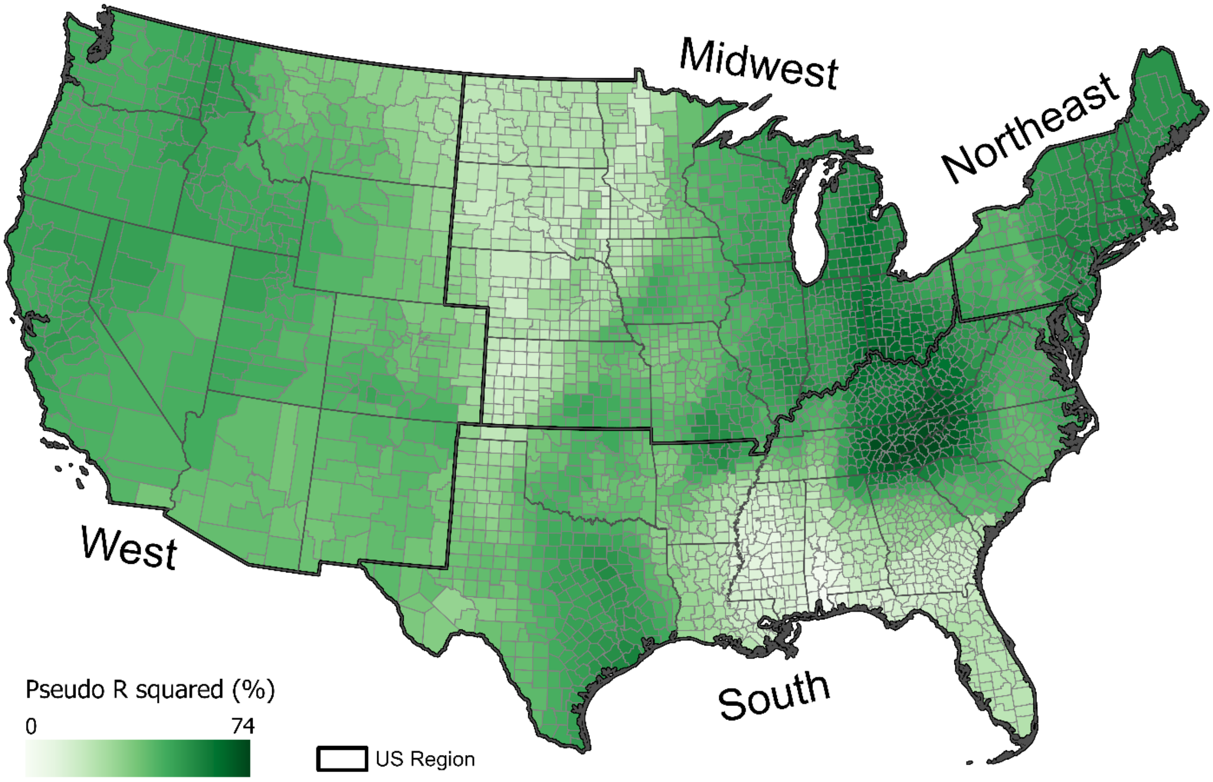

© 2022 Dong W et al. *JAMA Network Open.*

eFigure 4. Risk Factors Prevalence and Areas With High Variable Importance From the Geographical Random Forest (High Resolution Maps)

Notes: All variables use their own respective scales and are classified by quartile.

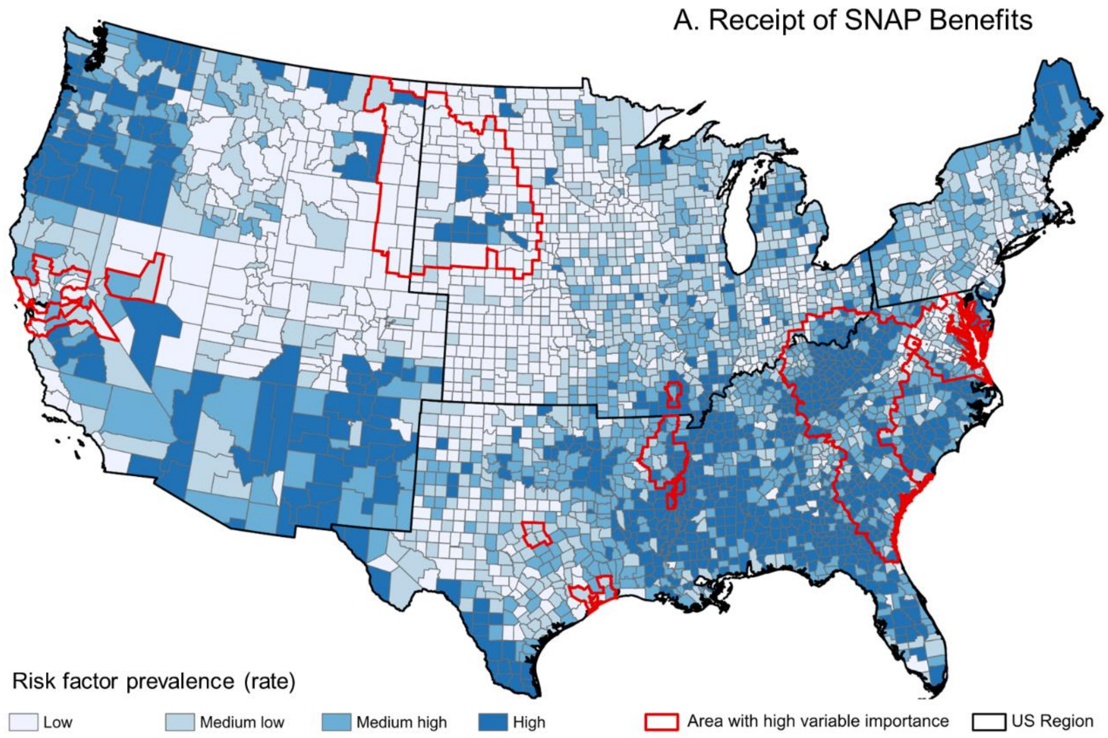

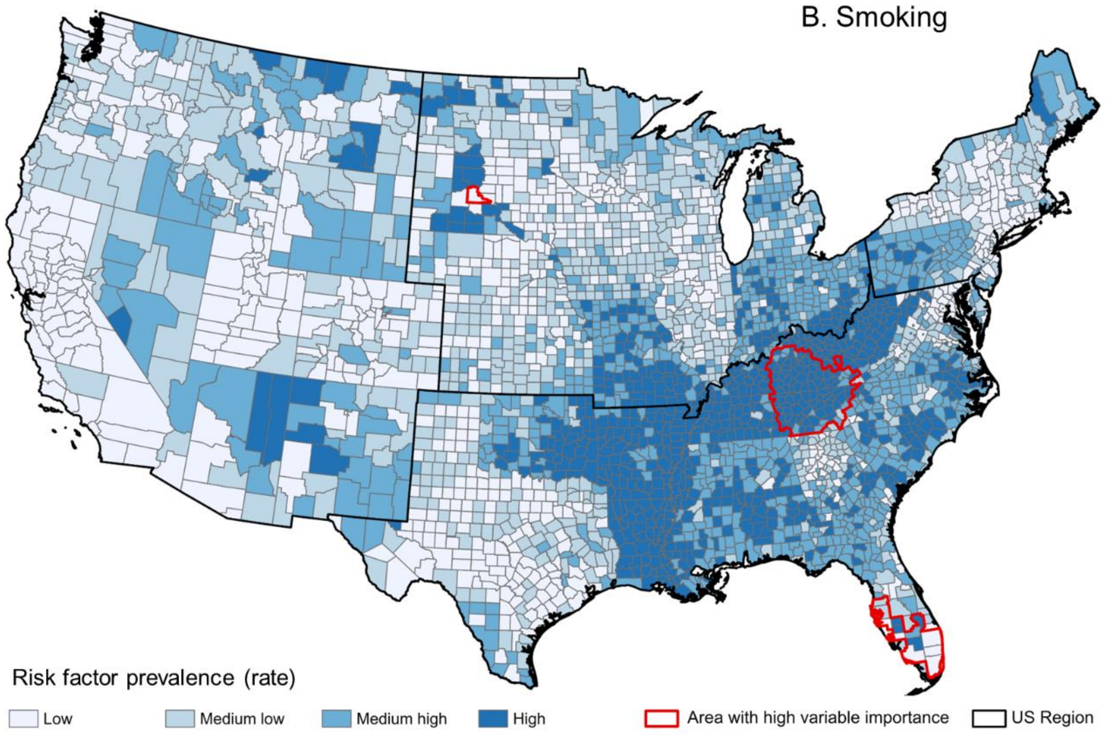

© 2022 Dong W et al. *JAMA Network Open.*

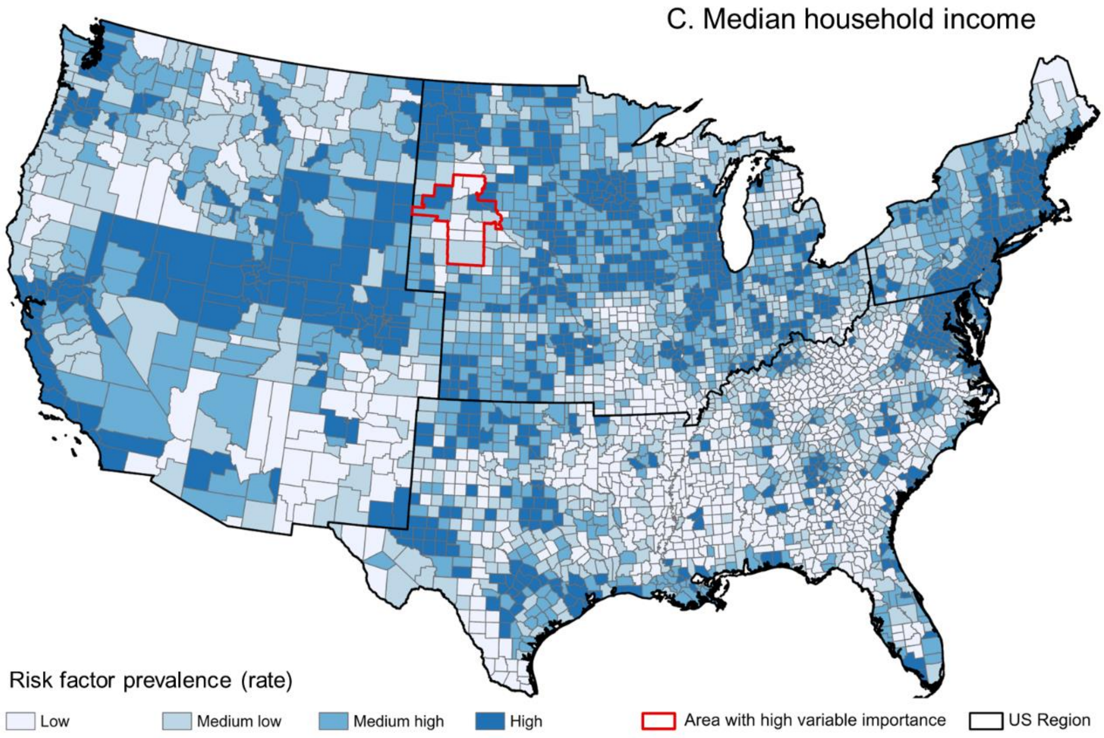

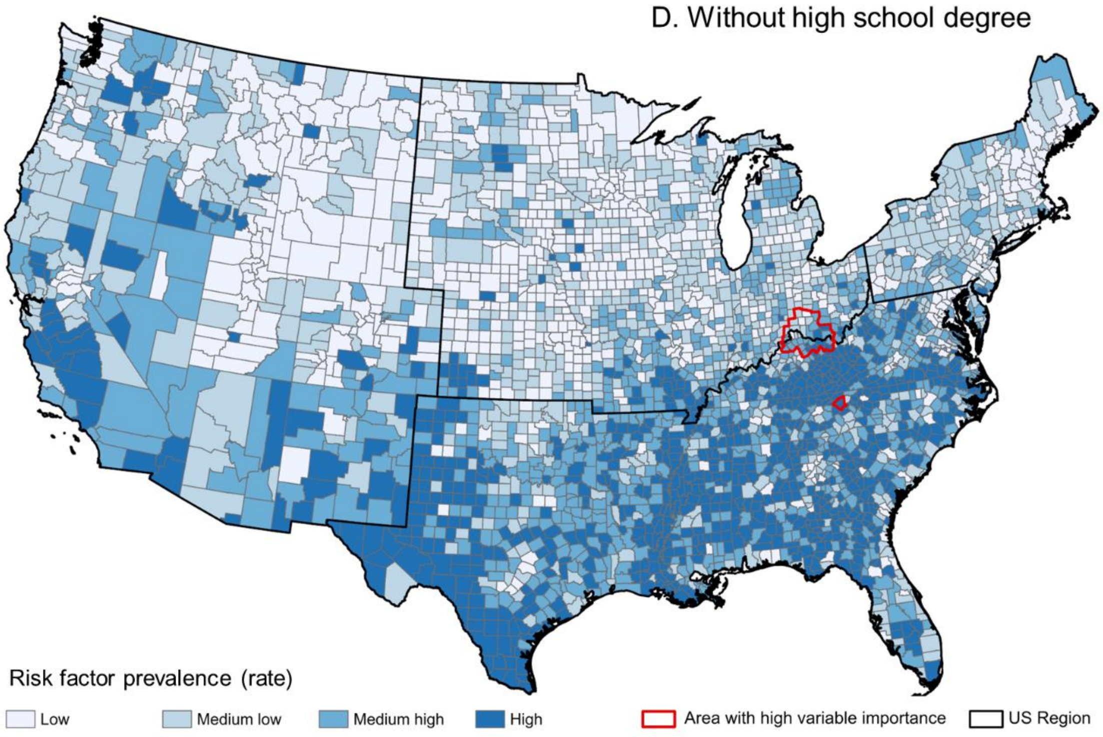

© 2022 Dong W et al. *JAMA Network Open.*

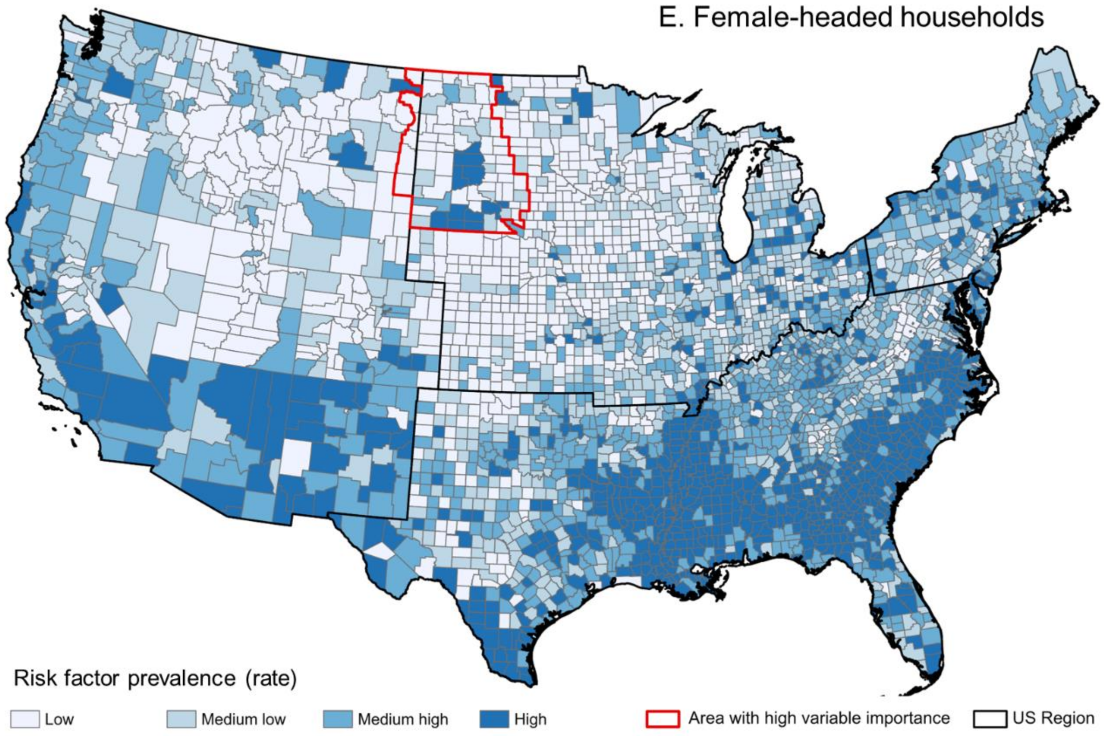

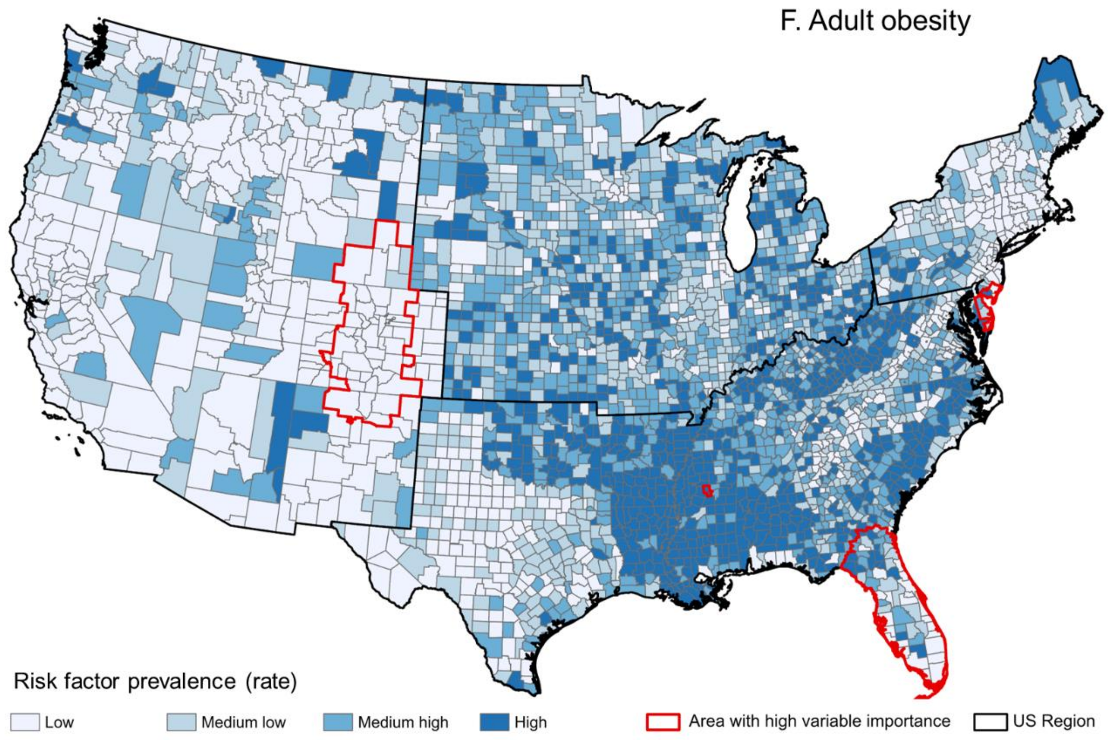

© 2022 Dong W et al. *JAMA Network Open.*
